# Enzyme Replacement in Gaucher Disease

**DOI:** 10.1371/journal.pmed.0010021

**Published:** 2004-11-30

**Authors:** Ernest Beutler

## Abstract

The development of enzyme replacement therapy for Gaucher disease was a triumph of translational medicine. What were the key steps in its development? What are the controversies surrounding its use?

Gaucher disease is the most common lysosomal storage disorder (Box 1). A deficiency of the enzyme glucocerebrosidase (Figure 1) causes accumulation of the glycolipid glucocerebroside in macrophages throughout the body. In the viscera, glucocerebroside arises mainly from the biodegradation of red and white blood cells. In the brain, glucocerebroside arises from the turnover of complex lipids during brain development and the formation of the myelin sheath of nerves. The disease may be discovered as an incidental finding in the elderly because of mild thrombocytopenia or splenomegaly, or it may present early in life with hepatosplenomegaly, thrombocytopenia, anemia, and bone lesions.

**Box 1. What Is Gaucher Disease?**

Gaucher disease is an inherited metabolic disorder in which harmful quantities of a fatty substance called glucocerebroside accumulate in the spleen, liver, lungs, bone marrow, and, in rare cases, the brain. There are three common forms.

Type 1 is the most common. Clinical features include easy bruising, anemia, low blood platelets, enlargement of the liver and spleen, bone disease, and, in some instances, lung impairment. There are no signs of brain involvement. Problems may begin early in life, be delayed until adulthood, or not occur at all.

In type 2, liver and spleen enlargement are apparent by three months of age, and there is extensive and progressive brain damage. These patients usually die by two years of age.

In type 3, liver and spleen enlargement is variable, and signs of brain involvement, such as seizures, become apparent gradually.

**Figure 1:**
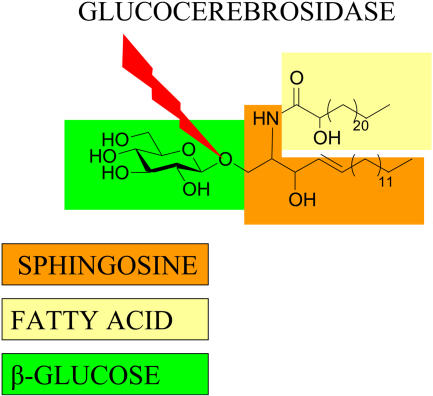
Glucocerebrosidase Cleaves a Linkage within Glucosylceramide, a Normal Intermediate in Glycolipid Metabolism

Until 1990, treatment consisted only of palliative measures such as splenectomy and hip replacement. The development of enzyme replacement therapy for Gaucher disease, that is, exogenous administration of the missing enzyme, is a triumph of translational medicine. At the same time, powerful commercial interests may have been influential in physicians adopting a high-dose rather than a low-dose treatment schedule. Moreover, the high cost of enzyme replacement therapy forces us to consider what society can afford in the way of palliative treatments for very rare diseases.

## The History of Enzyme Replacement Therapy

The possibility that the therapeutic replacement of enzymes missing from lysosomes could be achieved was first raised by de Duve forty years ago when he wrote: “Any substance that is taken up intracellularly by an endocytic process is likely to end up within lysosomes. This obviously opens up many possibilities for interaction, including replacement therapy” [1].

Type 1 Gaucher disease, the most common type, seems a particularly suitable target for enzyme replacement therapy because of the lack of central nervous system involvement (visceral damage in Gaucher disease is reversible whereas the brain damage usually is not). By the 1970s, the underlying enzyme deficiency had been identified, and methods had been developed to purify the enzyme from human placenta in a high state of purity. Three groups of investigators then attempted to treat the disease by infusing exogenous enzyme.

In the United States, at the National Institutes of Health in Bethesda, Maryland, the unaltered enzyme was infused directly into the venous circulation [2]; at City of Hope in Duarte, California, it was entrapped in red cell membranes coated with antibody in an effort to direct it to macrophages [3]. In Harrow, United Kingdom, the enzyme was delivered entrapped in liposomes [4]. Although some mildly encouraging results were achieved, it was clear that none of these approaches was likely to be translated into a useful treatment.

The needed conceptual breakthrough was provided by the identification of a mannose receptor on macrophages and the suggestion that this might prove useful in replacement therapy for Gaucher disease [5]. This led to the development of a modified enzyme, processed to expose mannose, and to its production on an industrial scale from placentas. After the gene encoding the enzyme was cloned [6], a recombinant product became available.

## The Pivotal Study

The first study of commercially produced mannose-enriched glucocerebrosidase was carried out in Bethesda, Maryland, on only 12 patients, presumably because of a limited supply of the enzyme [7]. Given this small cohort of patients, only a single dose (60 units/kg) was administered. This dose was given every two weeks to ten of the patients, while two patients received it weekly. This is manifestly an unusual dose schedule for a preparation with a circulating half-life of only about 12 min that is being targeted to a relatively small number of receptors. Many of the patients studied did not live near Bethesda, and it is likely that the dose schedule that was chosen was based on convenience rather than on sound pharmacokinetic principles. Since it was unlikely that a second study would be launched if the first failed, the investigators wisely used a very generous dose of enzyme to maximize the probability that the trial would be successful. Intravenous administration of the enzyme produced objective clinical improvement (such as reduced liver and spleen size and increased hemoglobin levels and platelet counts).

The enzyme was promptly approved and marketed. Since only a single dose had been tested, this was the dose that most physicians administered in clinical practice. But the preparation was extremely costly—about US$4.00 per unit. At the dose used in the pivotal trial, a 70-kg patient would receive enzyme costing US$16,800 every two weeks.

## Dosage Considerations

### Visceral organ responses.

But was the large dose given actually the dose required? There were no data, and many physicians were unwilling to give less than the dose that had been used in the pivotal trial. Moreover, since most physicians took care of only one or at most two patients with the disease, they were not in a position to perform a dose-ranging study. And industry had no interest in supporting studies to show that a lower dose yielded equivalent results.

**Figure 2:**
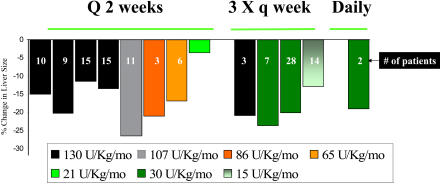
A Meta-Analysis of the Decrease in Liver Size in Patients with Gaucher Disease Documented for Various Doses of Enzyme Given As Replacement Therapy The individual studies included in the meta-analysis are listed in [9].

But clinical trials carried out in our National Institutes of Health–sponsored General Clinical Research Center quickly established that a quarter of the dose given at more frequent intervals was fully effective [8]. By 2000, a considerable body of data had accumulated, making it possible to perform meta-analyses of the relationship between the total monthly dose, the interval at which the dose is administered, and the decrease in the size of the liver. The results were clear (Figure 2) [9]. Even a dose of only 15 units/kg/mo, one-ninth of the dose given in the pivotal trial, resulted in an excellent clinical response. Most patients were receiving a substantial overdose of an extremely costly preparation. The data indicate that when very large doses are administered, the two-week time interval is adequate to give an optimal response, but when more modest doses are administered, more frequent infusions greatly improve the response [9].

Recent “consensus recommendations,” which were supported in part by the Genzyme Corporation, the manufacturers of recombinant human glucocerebrosidase (imiglucerase, brand name Cerezyme), suggest that children be given an initial dose of 30 to 60 units every two weeks [10]. But there is no high-quality evidence that such a costly treatment regimen provides results superior to those achieved with smaller doses. The only support for recommending this high dosage comes from uncontrolled studies showing that in some children bone lesions may progress at low dosages. However, we know from our own published observations that skeletal progression and even fractures also occur in some individuals receiving high-dose therapy [11]. Thus, I would caution against any recommendations to give high-dose therapy that have not been based on well-designed randomized, controlled trials. Having said this, I recognize that most, but not all, of the patients that were included in our meta-analyses were adults, whereas the company-sponsored consensus recommendations refer to children. However, in the absence of any evidence-based rationale for administering large, costly doses of enzyme, I believe that the use of smaller, more frequent doses is the most prudent treatment approach.

**Figure 3:**
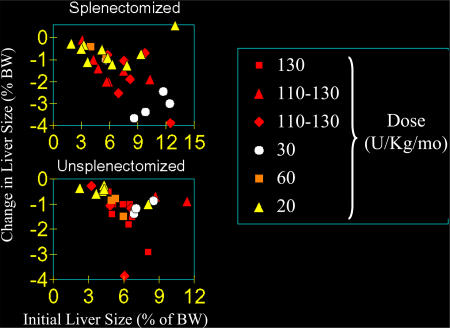
A Meta-Analysis of the Decrease in Liver and Spleen Size in Patients with Gaucher Disease As a Function of the Initial Organ Size BW, body weight. Redrawn from [11].

It is often assumed that patients with severe disease require larger doses of enzyme than those with mild disease, but a meta-analysis based on liver size or spleen size made it clear that this is not the case (Figure 3) [12]. Large organs shrink more rapidly than smaller ones, and this is true regardless of the dose that is used [13].

### Skeletal response.

The response of enlarged viscera to enzyme infusion is much more rapid than the response of bones. In one early study, the large dose used in the pivotal trial was given for up to four years to patients with bone disease, and although the response was slow, gradual improvement occurred [14]. Strangely, the authors concluded that large doses were required—“strangely,” because they did not give smaller doses to any patient. Subsequently, it was shown that less than a quarter of the dose (only 30 units/kg/mo) produced an equivalent response [15].

## Whom to Treat

The severity of Gaucher disease is very variable. We have estimated that some 60% of patients homozygous for the common *c.1226 C → G (N370S)* mutation never come to medical attention [16]. Accordingly, many—possibly most—patients with Gaucher disease require no treatment. In adults, the disease is rarely progressive [11,17]. What you see is what you have, more or less. Bone fractures, of course, are not gradual events but sudden ones. But almost invariably they occur in patients who already have very substantial, demonstrable bone disease. In children, the situation is different, and progression is common. It is only with proper awareness of the natural history of the disease that one can make rational judgments regarding who needs treatment.

## Individualized Treatment

Evaluating dose–response relationships in patients with Gaucher disease has been difficult for several reasons. The number of new patients requiring therapy is relatively small, and the Genzyme Corporation has done little to encourage the performance of dose–response studies, making it difficult to enroll patients. But beyond that, the response of patients to any dose is variable. Some authors have suggested that this may be due to individual differences in dose requirements—that some patients are relatively resistant and require a large dose, while others do well on a small dose [18]. This is an attractive concept, but is it correct? Another meta-analysis indicates that it is not. Rather, there are patients who respond poorly to any dose and others who respond well to any dose [19]. Moreover, quadrupling the dose does not increase the rate of response [11].

## What Does the Future Hold?

The quality of life for patients with Gaucher disease has been greatly improved by the development of enzyme replacement therapy. Manufacturing and selling the enzyme has also been enormously profitable for industry. This profitability has served as a stimulus for the development of enzyme replacement treatments for diseases less common and generally less responsive to treatment than Gaucher disease. Given the small target population, these treatments are enormously costly on a per-patient basis. Treatments for Fabry disease and Hurler-Scheie disease (also called mucopolysaccharidosis I) are already licensed, and others are on the way [20,21,22]. This brings us face-to-face with a major ethical dilemma. We do not put a price on human life. Yet health-care resources are a zero-sum game. What is spent on one disease cannot be spent on another. Is it better to treat one child with Hurler-Scheie disease [22] or to provide good prenatal care to 100 women who might not otherwise obtain it, or for that matter, to feed 1,000 malnourished children? These are difficult decisions that will be forced on us as enzyme replacement and other high-technology therapies come of age.
